# Anion Exchange Affinity-Based Controllable Surface Imprinting Synthesis of Ultrathin Imprinted Films for Protein Recognition

**DOI:** 10.3390/polym14102011

**Published:** 2022-05-14

**Authors:** Renyuan Song, Xiaofeng Yu, Muxin Liu, Xiaoling Hu, Shengqing Zhu

**Affiliations:** 1 School of Materials and Chemical Engineering, Bengbu University, Bengbu 233030, China; yuxfchemistry@163.com (X.Y.); schemistry01@163.com (M.L.); yuxfchem@126.com (S.Z.); 2 Bengbu Product Quality and Inspection Institute, Bengbu 233030, China; 3 School of Chemistry and Chemical Engineering, Northwestern Polytechnical University, Xi’an 710129, China; shichemistry@126.com

**Keywords:** anion exchange affinity-based interaction, surface-initiated photoiniferter-mediated polymerization, immobilization template, protein recognition

## Abstract

Anion exchange affinity-based controllable surface imprinting is an effective approach to overcome low imprinting efficiency and high non-specific binding capacity. The template proteins were first immobilized on the anchored tetraalkylammonium groups of the nanoparticles via anion exchange affinity-based interactions, enabling monolayer sorption using a low template concentration. The combined use of surface-initiated photoiniferter-mediated polymerization to precisely control the imprinted film thickness, allowing the formation of homogeneous binding cavities, and the construction of effective binding sites resulted in a low non-specific binding capacity and high imprinting efficiency. The obtained imprinted films benefited from the anion exchange mechanism, exhibiting a higher imprinting factor and faster binding rate than the reference material. Binding tests revealed that the binding strength and selective recognition properties could be tuned to a certain extent by adjusting the NaCl concentration. Additionally, in contrast to the harsh template elution conditions of the covalent immobilization approach, over 80% of the template molecules were readily removed from the imprinted films using supersonic elution with an aqueous mixture of NaCl and HAc. Introducing template immobilization by anion exchange interactions to the synthesis of imprinted materials may provide a new approach for effective biomacromolecular imprinting.

## 1. Introduction

Molecular imprinting is a technique that is used to generate artificial receptors with specific binding sites; the receptors are tailor-made in situ by the copolymerization of functional monomers and cross-linkers in the presence of a target template [1,2]. Compared with natural recognition materials (e.g., antibodies and enzymes), molecularly imprinted polymers (MIPs) offer several advantages (e.g., favorable mechanical, thermal, and chemical stability, as well as low cost) [3]. To date, MIPs have already been successfully applied in separation [4], sensors [5], and catalysis [6]. Although the imprinting of small molecules is straightforward [7], imprinting large and complex molecules (e.g., proteins [8], enzymes [9], DNA [10], and viruses [11]) remains a challenge.

The primary issue in protein imprinting is the large molecular size of proteins, which limits their mass transfer across the highly cross-linked polymeric matrix, restricting the ease of template protein removal and uptake [12]. The flexible conformation and complex structure of proteins necessitate the implementation of the imprinting process in a stable and mild aqueous environment. Moreover, the formation of highly selective imprinted cavities is challenging because of the complexity of the functional sites in the template proteins. A variety of strategies have been developed to overcome the mass transfer limitation, such as epitope imprinting [13] with a fragment of the original protein molecule as a template, the use of moderately cross-linked hydrogels [14], and surface imprinting [15]. Thus, imprinting materials with sites situated at or close to the surface of the polymeric matrix, enabling easy access to target protein molecules, is considered the most promising approach for protein templates [16]. Nevertheless, random rotation of the template proteins spontaneously occurs during the imprinting process, resulting in the formation of various complex imprinting cavities at random positions in the polymers. Consequently, the obtained imprinted materials exhibit a lower binding affinity and selectivity toward the template proteins [17].

The immobilization of proteins has recently been widely studied, motivated by the prospect of using polymeric or inorganic particles as new types of substrate materials [18,19]. In this strategy, proteins are immobilized on surfaces using both covalent and non-covalent approaches. Covalent immobilization has been achieved by forming covalent bonds between the template proteins and substrate particles [20,21,22]. Shiomi et al. [21] pioneered a strategy based on covalent template protein immobilization using aldehyde-group-modified silica particles as substrates. This protocol was further developed by Bonini et al. [22] to fabricate silica-based imprinted particles using APBA instead of modified silica particles. However, this approach has a considerable drawback with respect to the harsh conditions required for removing the template, which could affect the stability unless the template is stripped after sacrificing the substrate. The noncovalent immobilization method mainly relies on reversible physical adsorption and ionic linkages via stable ionic bonds [23]. The physical adsorption method was developed by Fu et al. [24] to immobilize template proteins on the surfaces of carboxylic acid-modified silica nanoparticles. An ultrathin imprinted film was then synthesized through surface polymerization at a low monomer concentration. The resulting imprinted particles demonstrated a high imprinting factor and specific rebinding capacity for the template. However, template leakage from the substrate may occur when the interactions are relatively weak in the imprinting process, leading to the decimation of the template and low imprinting efficiencies. Inspired by such a template immobilization strategy, we propose the introduction of tetraalkylammonium (TAA) groups as a substitute for carboxylic acids at the nanoparticle surface as a strategy to overcome template leakage and achieve a high immobilization capacity. The TAA groups can adsorb the template proteins and form stable ionic bonds via an anion exchange reaction. The resulting surface-initiated controllable polymerization can be utilized to precisely control the imprinted film thickness, allowing the generation of homogeneous binding cavities and construction of effective binding sites situated at or close to the substrate surface with fast mass transfer rates, a high imprinting efficiency, and enhanced selectivity toward the template.

In this study, nanoparticle substrates with surface-bound dithiocarbamte and benzyl groups were first synthesized via two-stage dispersion polymerization and then chemically modified to fabricate TAA groups on the substrate surface. The anchored TAA groups were expected to play a crucial role in immobilizing the template proteins through anion exchange affinity-based interaction [25]. Then, a series of modified procedures were performed to immobilize the template proteins on the substrate surface, monitored by X-ray photoelectron spectroscopy (XPS) and Fourier transform infrared spectrometry (FT-IR). Subsequently, a simple surface-initiated photoiniferter-mediated polymerization (SI-PIMP) was carried out to generate unified ultrathin imprinted films over the modified nanoparticles. Finally, the template proteins were removed by ultrasonic cleaning with an HAc solution containing NaCl. Complementary binding sites were created for the template proteins on the nanoparticle surface. The resultant imprinted nanoparticles were characterized using scanning electron microscopy (SEM), transmission electron microscopy (TEM), and nitrogen gas sorption measurements. The binding characteristics of the imprinted nanoparticles were evaluated using batch binding and selective binding tests, as well as binding kinetics studies.

## 2. Experimental

### 2.1. Materials

4-Chloromethylstyrene (4-CMS) and divinylbenzene (DVB) were obtained from Shanghai TCI. *N*,*N*-methylenebisacrylamide (MBAA) and acrylamide (AAm) were purchased from Alfa. The samples were then distilled under vacuum to remove the inhibitor. 4-Vinylbenzyl *N*,*N*-diethyldithiocarbamate (VBDC) was prepared following a procedure reported in [26]. The Shanghai Chemical Corporation supplied polyvinylpylrrolidone-30 (PVP-30) and azobisisobutyronitrile (AIBN). Trimethylamine (TMA) and ethanol were obtained from the Sinopharm Chemical Reagent Co., Ltd. Bovine serum albumin (BSA; MW 66.4 kDa, pI 4.7), ovalbumin (OVA; MW 45 kDa, pI 4.7), hemoglobin (Hb; MW 64.5 kDa, pI 6.8–7.0), and lysozyme (Lys; chicken egg white, MW 14.4 kDa, pI 10.7) were purchased from Sigma-Aldrich. DVB and 4-CMS were purified by passing them through a column filled with basic aluminum oxide to remove the inhibitor and were stored at −5 °C. AIBN was recrystallized in ethanol. The other chemicals were used as received without further purification.

### 2.2. Characterization

The morphologies and structures of the samples were determined by SEM (FEI, Quanta 600FEG, USA) and TEM (FEI, Tecnai G2 F30, S-TWIN, USA). XPS measurements were carried out with an ESCALab220i-XL electron spectrometer from VG Scientific (UK) using 300 W Al Kα radiation. The FT-IR measurements were recorded on a WQF-310 Fourier transform spectrophotometer (Shimadzu, Kyoto, Japan). Nitrogen adsorption tests were performed using an ASAP 2020 surface area and porosity analyzer (Micromeritics, USA). Adsorption data were obtained using a UV-2450 spectrophotometer (Shimadzu, Kyoto, Japan). Finally, the ζ potential of the polymers was measured using a Zetasizer Nano Z (Malvern Instruments, Ltd., Malvern, UK) in phosphate buffer solution (PBS).

### 2.3. Preparation of Substrate Nanoparticles

**Figure 1:**
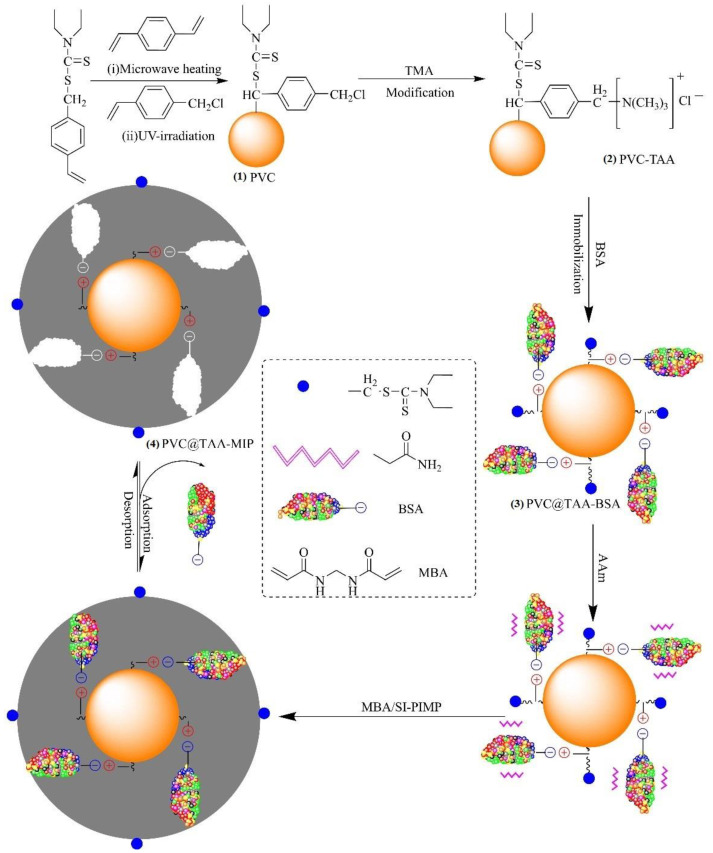
BSA-imprinted thin films were synthesized via anion exchange, affinity-based, controllable, surface imprinting approach.

Cross-linked poly (VBDC-CMS) nanoparticles (denoted as PVC) with surface-bound dithiocarbamte and chloromethyl groups were synthesized by dispersion polymerization. The typical procedure was as follows: a mixture of ethanol (95 mL), deionized water (5 mL), and PVP-30 (0.8 g) was placed in a 250 mL, four-neck, round-bottom, quartz flask equipped with a nitrogen inlet tube and mechanical stirrer. Then, a mixture of DVB (0.5 mL, 3.53 mmol), VBDC (3.5 g, 15.44 mmol), and AIBN (0.06 g, 0.37 mmol) was added to the flask and bubbled with N_2_ for 30 min. After the flask was placed in a microwave-ultraviolet–ultrasonic trinity synthesis extraction apparatus (MUUTSEA; UWave-1000, Shanghai, China), the polymerization reaction was performed using microwave heating (180 W) and magnetic stirring at 200 rpm for 3 h. After polymerization, the solution was cooled to room temperature, and then a mixture of 4-CMS (0.5 mL, 3.55 mmol) and ethanol (4.5 mL) was slowly added to the flask. Subsequently, grafting polymerization was conducted using ultraviolet radiation provided by a high-pressure mercury lamp (300 W, 365 nm) while stirring at room temperature for 30 min. After graft polymerization, the obtained polymers were washed several times with methanol to remove the unreacted monomer and free stabilizer. Finally, the as-prepared PVC nanoparticles were dried at 40 °C under a vacuum for 24 h to obtain a white powder (Figure 1).

### 2.4. Immobilization of Template Protein

The anchored TAA groups of the PVC nanoparticles (denoted as PVC@TAA) were synthesized using the method reported by Pepper et al. [27]. TMA (2.5 g) was added to a mixture of ethanol (50 mL) and PVC nanoparticles (1.0 g), and irradiated with ultrasound waves at 50 °C. The reaction was continued for 36 h. The final light-yellow nanoparticles (Figure 1(2)) were rewashed several times with deionized water and ethanol to remove the solvent and free TMA, followed by drying under a vacuum at 40 °C for later use. Similar to PVC@TAA, the anchored hydroxyl groups of the PVC nanoparticles (denoted as PVC@OH) were prepared using chemical modification with NaOH. The final product was dried under a vacuum at 40 °C for subsequent use.

PVC@TAA nanoparticles (0.10 g) were added to a mixture of BSA (6.37 mg, 0.096 µmol) and 10 mM PBS (pH 7.0, 80 mL), which was magnetically stirred at 100 rpm for 2 h at 5 °C to allow coupling to occur. Then, the obtained products were rinsed several times with 10 mM PBS (pH 7.0, 5 mL) to remove loosely bound template proteins, and all the elutions were collected as a residual solution. Finally, the final white (Figure 1(3)) nanoparticles (denoted as PVC@TAA-BSA) were dried under a vacuum at a low temperature for future use. By utilizing PVC@OH instead of PVC@TAA, the corresponding PVC@OH-BSA nanoparticles were obtained under the same preparation conditions.

### 2.5. Synthesis of BSA-Imprinted Films

The method reported by John was used to synthesize unified ultrathin imprinted films through SI-PIMP [28]. The typical procedure was as follows: PVC@TAA-BSA nanoparticles (0.1 g) were dispersed in 10 mM PBS (pH 7.0, 20 mL) by ultrasonic vibration (150 W). Then, AAm (1.2 mmol, 0.0853 g) and MBAA (2.4 mmol, 0.3700 g) were dissolved in 10 mM PBS (pH 7.0, 20 mL) and added to the aforementioned solution. After 1 h of shaking for preassembly, the reaction mixture was transferred to a 100 mL, four-neck, round-bottom, quartz flask and purged with N_2_ for 30 min. The flask was placed in the MUUTSEA, which made precisely controlling the magnetic stirring speed and temperature of the reaction system possible. The polymerization reaction was performed using ultraviolet radiation (300 W, 365 nm) with magnetic stirring at 100 rpm and 0 °C for 30 min. After polymerization, the resulting products were washed several times with deionized water until the supernatant was clear. NaCl (0.1 M, 10 mL) was eluted to remove the template protein until BSA residues were no longer observed. Finally, the obtained BSA-imprinted polymer particles (denoted as PVC@TAA-MIP, Figure 1(4)) were dried in a vacuum at a low temperature (0 °C) for 36 h.

The corresponding non-imprinted nanoparticles (denoted as PVC@TAA-NIP) were prepared under the same preparation conditions, except without surface immobilization of the template protein before the polymerization of the external shell layer. The obtained products were used as non-imprinted control nanoparticles for comparison in subsequent experiments. 

### 2.6. Removal of Template Protein

The template protein was washed using ultrasound vibration (300 W) with different ratios of the NaCl–HAc solution at room temperature for 3 h and eluted over time. For each elution, 100 mL of the NaCl–HAc solution was used. The elution was considered complete when the change in the removal of the template protein was less than 0.1%. Then, 1 L of deionized water was used to remove all traces of NaCl and HAc. The template protein (BSA) removal from the imprinted films was confirmed using a UV spectrophotometer at 278 nm. Finally, the elution of the corresponding PVC@TAA-NIP nanoparticles was carried out in the same manner.

### 2.7. Binding Experiments

The batch rebinding tests involved the rebinding of isotherms, selective rebinding, competitive rebinding, and the rebinding dynamics. In these tests, approximately 10 mg batches of particles were incubated in 10 mL protein solutions with various concentrations. After incubation, each protein solution was collected, and the concentration was determined using a UV spectrophotometer according to our previous studies [29]. The amount of protein adsorbed by the particles was calculated according to the following equation:

(1) $$Q_{e}=\frac{(C_{0}-C_{e})V}{m},$$

where *C*_0_ (mg·mL^−1^) and *C_e_* (mg·mL^−1^) represent the initial and final concentrations, respectively; *V* (mL) is the total volume of the adsorption mixture; and *m* (g) is the number of particles. All the tests were conducted in triplicate. The specific recognition properties of the imprinted particles were evaluated using the imprinting factor (*IF*), which is defined as *IF* = *Q*_MIP_/*Q*_NIP_, where *Q*_MIP_ and *Q*_NIP_ are the adsorption capacities of the imprinted and non-imprinted polymers, respectively. The selectivity factor (*β*) is defined as *β* = *IF*_tem_/*IF*_ana_, where *IF*_tem_ and *IF*_ana_ are the imprinting factors for the template molecule and analog, respectively. Rebinding isotherm studies were carried out using 10 mg batches of particles at various concentrations. The particles were first incubated in PBS (pH 7.0, 10 mM) at 25 °C for 10 min, and then the equilibrium solutions were replaced with 10 mL volumes of the same buffer solution with various initial concentrations of BSA (0–1.5 µM) for 120 min. The amount of protein adsorbed by the particles was then determined as described here.

The selectivity of the particles toward proteins was investigated to compare the adsorption of the template with that of the reference proteins. A known mass of dry particles was first incubated in PBS (pH 7.0, 10 mM) at 25 °C for 10 min, and then the equilibrium solution was replaced by 10 mL of the same buffer solution containing 0.75 µM of the template or reference proteins. Next, the solution was shaken for 120 min at 25 °C. The amount of protein adsorbed by the particles was determined as described earlier. The UV detection wavelengths were fixed at 278 nm for BSA, OVA, and Lyz and adjusted to 404 nm for Hb.

Hb was selected as the competitive protein. A competitive adsorption test was carried out using a binary solution of BSA and Hb with individual initial concentrations of 0.75 µM. The competitive adsorption experiment was performed in a manner similar to the aforementioned single-protein adsorption tests. The final concentrations of both proteins in the supernatant were determined using double-wavelength UV/vis spectrophotometry, with detection wavelengths of 278 and 404 nm for BSA and Hb, respectively.

## 3. Results and Discussion

In our previous study [29], a polymerizable ionic liquid (IL) was utilized as a functional monomer to synthesize molecularly imprinted anion exchange hydrogels with excellent affinity and a fast binding rate toward the BSA template. In addition, Row et al. [30,31] reported imprinted anion exchange polymers synthesized using other charged monomers. However, applying randomly oriented charged molecules as functional monomers could lead to the formation of charged moieties of non-imprinting active sites outside the imprinted materials, resulting in significant non-specific binding. In this study, anchored charged functional groups were used as active sites to immobilize the template through the anion exchange mechanism to ensure that binding sites were only formed inside the imprinted materials. In this approach, the residual charged moieties were completely embedded after polymerization. Consequently, non-specific binding from the residual charged moieties could be avoided, leading to an enhanced imprinting efficiency for the imprinted materials after the template was removed.

Herein, we report a novel and facile approach, anion exchange affinity-based controllable surface imprinting, for the efficient imprinting of BSA. This strategy is schematically presented in Figure 1. Poly(DVB-VBDC) nanoparticles with surface-bound dithiocarbamte groups were first synthesized by dispersion polymerization using microwave irradiation. Then, linear poly(4-CMS) was grafted onto the poly(DVB-VBDC) surface using photoiniferter polymerization. Subsequently, the obtained PVC nanoparticles were modified using TMA via a benzyl group reaction to introduce positively charged TAA at the substrate surface. Subsequently, the ionic state of the BSA molecules (pH 7.0, PI = 4.7) could be immobilized on the PVC@TAA surface through an anion exchange affinity-based interaction [32,33]. After adding the functional monomer (Aam) and cross-linker (MBAA) to generate a size and shape that is a complementary match to binding cavities with template proteins, the polymerization reaction was carried out using ultraviolet radiation under low-temperature conditions. During polymerization, the attached dithiocarbamte groups on the surface of the PVC nanoparticles acted as initiators for the surface-initiated activator generated by SI-PIMP. This approach allowed the precise control of the imprinted film thickness, where the cross-linked polymer matrix could be propagated from the multireactive nanoparticle substrate in proportion to the polymerization time [34]. Finally, imprinted nanoparticles were obtained by removing the template protein from the imprinted films using solvent extraction.

### 3.1. Surface Modification of Substrate Nanoparticles

**Figure 2:**
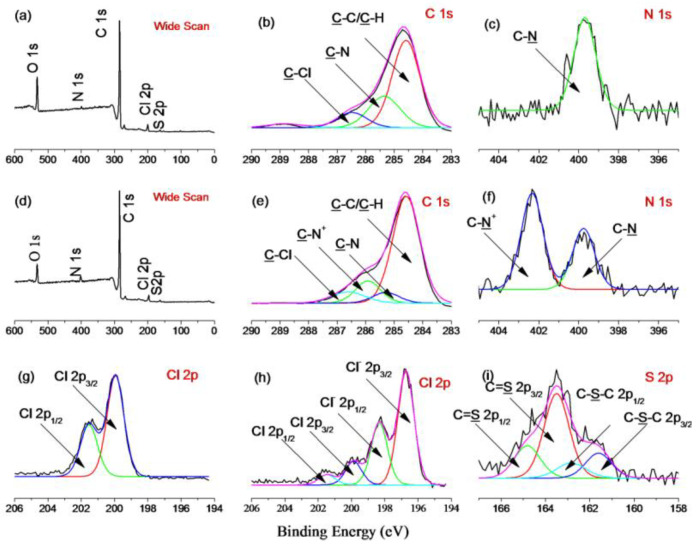
(**a**) Wide scan, (**b**) C 1s, (**c**) N1s, (**g**) Cl 2p, and (**i**) S 2p XPS results for PVC nanoparticles and (**d**) wide scan, (**e**) C 1s, (**f**) N1s, and (**h**) Cl 2p XPS results for PVC@TAA nanoparticles.

The introduction of TAA groups onto the surface of the PVC nanoparticles was verified by FT-IR and XPS analyses. In the spectrum depicted in Figure S1 (ESI †), the unmodified PVC has features at 1208 cm^−1^, corresponding to the C=S stretching vibration, and at 1415 and 1354 cm^−1^, corresponding to the C–H bending vibration of the dithiocarbamate group [35]. Further, the two peaks at 760 and 1264 cm^−1^ correspond to CH_2_–Cl asymmetric and symmetric stretching, respectively. Compared with PVC, PVC@TAA displays two new peaks at 1483 and 1377 cm^−1^, corresponding to the C–N stretching vibration and the C–H bending vibration of the TAA group, respectively [36]. Compared with the results of the XPS measurements for PVC and PVC@TAA, a new peak component with a binding energy of 286 eV, attributable to the C–N^+^ species, appears in the C 1s core-level spectrum of PVC@TAA (Figure 2e), whereas a new peak at a binding energy of 402.3 eV, ascribed to the C–N^+^ species [37], arises from the quaternization reaction between the benzyl groups and TMA in the N1s spectrum (Figure 2f). In addition, the Cl 2p core-level spectra consist of the Cl 2p_3/2_ and Cl 2p_1/2_ doublet at binding energies of approximately 196.7 and 198.3 eV, respectively, assignable to the ionic Cl (Cl^−1^) species of PVC@TAA [38]. By comparing the intensities of the C–Cl species in Figure 2h, which shows high intensities for the anionic chloride species at 196.7 eV (Cl 2p_3/2_) and 198.3 eV (Cl 2p_1/2_), it can be concluded that most benzyl groups of the PVC have been modified. Thus, these results confirmed that TMA was successfully coupled to the PVC surface.

**Figure 3:**
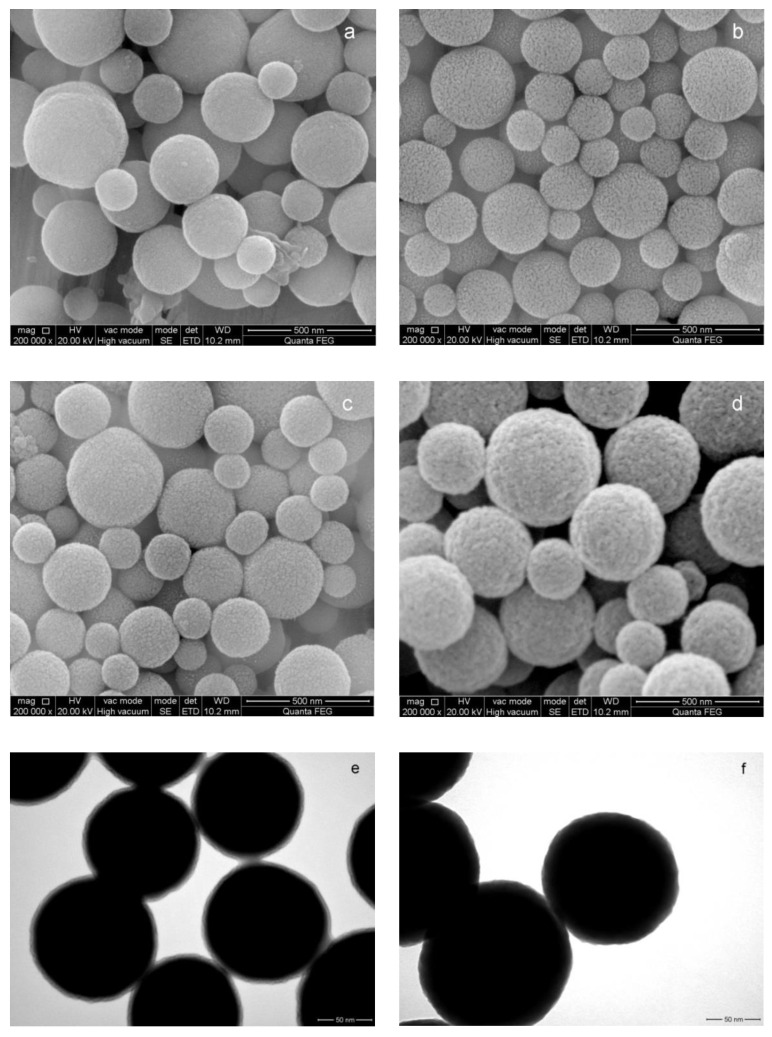
SEM and TEM images of (**a**) PVC, (**b**) PVC@TAA, (**c**,**e**) PVC@TAA-BSA, and (**d**,**f**) PVC@TAA-MIP.

The morphological structures of the PVC and PVC@TAA nanoparticles were characterized using SEM and BET. As shown in Figure 3, a smooth surface for the spherical PVC particles was observed in the SEM image (Figure 3a). However, it was noticeably different from the PVC@TAA surface (Figure 3b), because the latter demonstrated a rough surface with a substantial number of short holes, leading to a higher specific surface area (119.63 m^2^·g^−1^) than that of PVC (34.87 m^2^·g^−1^) (Table S1, ESI †). Porous nanoparticles with a high specific surface area can enhance the attachment via bio-affinity, enabling higher protein loading. Finally, the content of TAA groups was measured using the precipitation titration method and found to be 4.47 ± 1.38 µmol per gram of PVC@TAA.

### 3.2. Surface Immobilization of Template Proteins

**Figure 4:**
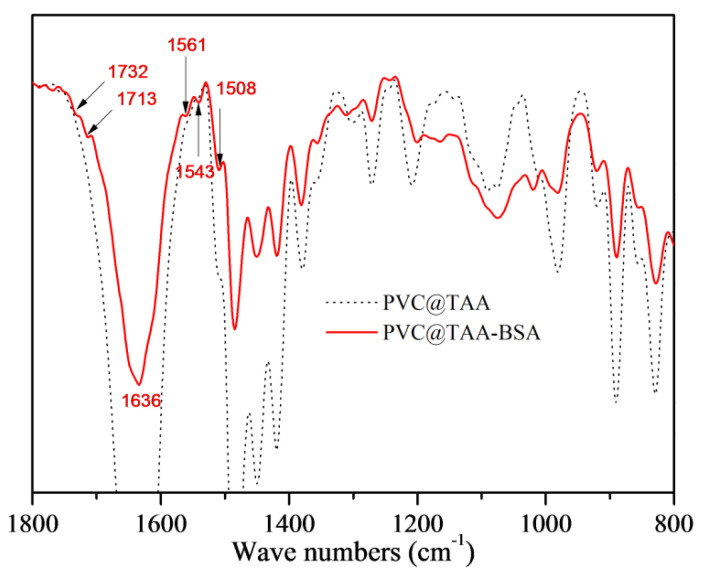
FT−IR spectra of PVC@TAA and PVC@TAA−BSA nanoparticles.

The immobilization of template proteins was monitored using FTIR and XPS. In the spectrum depicted in Figure 4, PVC@TAA-BSA displays two new peaks at 1732 and 1713 cm^−1^, corresponding to the C=O stretching vibration of the non-ionized and ionized carboxyl groups, respectively [39]. The new peak at 1636 cm^−1^ corresponds to an amide I band, whereas that at 1561 cm^−1^ is assigned to an amide II band. The bands at 1543 and 1508 cm^−1^ are designated to belong to different in-plane ring vibrations of the tyrosine groups in BSA [40]. The XPS spectrum for the immobilization layer was composed of C 1s and N 1s peaks (Figure S2, ESI †). After immobilization, two new peaks at binding energies of 288.6 and 400.1 eV were observed, which were attributed to the carboxyl (O=C–O) and amide/amino (O=C–NH/NH_2_) groups in BSA, respectively. More importantly, the peak of C–N^+^ at 286.0 eV (Figure 2e) shifted to a lower binding energy (285.7 eV, Figure S2b, ESI †), which could be attributed to the formation of ionic bonds via anion exchange affinity-based interactions between the carboxyl groups in the BSA and TAA groups at the PVC@TAA surface.

SEM and TEM further confirmed the immobilization of the templates. As observed from the SEM images of PVC@TAA nanoparticles (Figure 3c), the morphology of PVC@TAA-BSA exhibited a distinct structure, demonstrating a rough surface. This indicated that the template proteins were successfully immobilized on the surface of the PVC@TAA nanoparticles. According to the TEM images of PVC@TAA nanoparticles (Figure 3e), the thickness of the immobilized layer of BSA was approximately 7 nm (dry state), which was close to the BSA molecular dimensions (14 × 4 × 4 nm) [41]. This result confirmed the near-monolayer coverage of the immobilized layer. Our synthesis procedure utilized a low BSA concentration to control the immobilized layer thickness and ensure monolayer BSA immobilization on the PVC@TAA surface.

BSA solutions with various concentrations were used to immobilize the template proteins in PBS (pH 7.0, 10 mM). The ζ potentials of the PVC@TAA-BSA in PBS were determined using a Zetasizer Nano Z instrument. The immobilization capacity of BSA was calculated according to Equation (1), and the results are summarized in Table S2 (ESI †). The ζ potentials and immobilization capacities of PVC@TAA-BSA exhibited significant variation with an increase in the concentration of the BSA solution. Remarkably, the ζ potential of PVC@TAA-BSA was reversed from positive to negative (+6.28 ± 0.21 mV to −11.29 ± 0.55 mV) when the concentration of the BSA solution changed from 3.6 to 4.8 µM. This result confirmed that the surface of PVC@TAA was entirely covered, providing an immobilization capacity of 0.992 ± 0.0363 µmol per g of PVC@TAA. Thus, a 4.8 µM BSA solution was employed to prepare the subsequent imprinted polymers. In contrast, the PVC@OH nanoparticles, as a platform to immobilize BSA via hydrogen bonding interactions, exhibited a low immobilization capacity (0.132 ± 0.0091 µmol per g of PVC@OH). This suggested that the high capacity of PVC@TAA corresponded to robust anion exchange affinity-based interactions.

### 3.3. Synthesis of BSA-Imprinted Thin Films

In the proposed molecular imprinting method, an external imprinted film was synthesized over PVC@TAA-BSA during the second-stage SI-PIMP with AAm and MBAA as the functional monomer and crosslinker, respectively. As shown in Figure S1 (ESI †), two significant bands at 1731 and 1638 cm^−1^, corresponding to the C=O stretching modes of MBAA and AAm, respectively, indicated the successful growth of covalently bonded polymers (MBAA) on the surface [42]. Further, the peak at 1208 cm^−1^, corresponding to the C=S stretching vibration of the dithiocarbamate group, is also observed in Figure S1 (ESI †). In addition, the XPS spectrum analysis showed a new binding energy of 288.8 eV (Figure S2e, ESI †), which could have been derived from the O=C-NH bonds, indicating the deposition of a poly(AAm-co-MBAA) thin film on the surface of the substrate. The S 2p core-level spectrum (Figure S2g, ESI †) consisted of the S 2p_3/2_ and S 2p_1/2_ doublet at approximately 161.6, 162.8, 163.5, and 164.9 eV [43], which could be attributed to the dithiocarbamte groups at the polymer surface, revealing that the imprinted thin films were successfully polymerized over the substrate surface by SI-PIMP. In addition, peaks corresponding to C–N^+^ (Figure S2e, ESI †), and C–N^+^ (Figure S2f, ESI †) showed no signal in the corresponding core-level spectrum, confirming that the absence of residual TAA groups on the surface could endow the imprinted films with a weak non-specific binding property.

The size and surface morphology of the PVC@TAA-MIP nanoparticles were examined using SEM and TEM. As can be seen in Figure 3d, the PVC@TAA-MIP had a rough surface as a result of the cross-linking reaction and removal of the template proteins by the elution solution. In addition, the TEM image of PVC@TAA-MIP (Figure 3f) shows that the unified ultrathin polymer film had a thickness of approximately 15 nm. This suggests that the imprinting effect may be further enhanced by controlling the shell layer thickness via the SI-PIMP reaction. The specific surface areas (Table S1, ESI †) of PVC@TAA-MIP and PVC were determined to be 104.59 and 83.41 m^2^·g^−1^, and the average pore diameters were 9.57 and 14.63 nm, respectively. PVC@TAA-MIP had a significantly large specific surface area, which might have been due to the large number of imprinted cavities located on the outside surface. Furthermore, the average pore diameter of the PVC@TAA-MIP satisfied the release and rebinding transfer requirements of the template.

Based on the above discussion, the successful preparation of a PVC@TAA-MIP core–shell structure using an immobilized template technique and surface-initiated polymerization could be confirmed by the complete characterization provided through SEM, TEM, BET, FT-IR, and XPS analyses. Additionally, after polymerization, the supernatant had no clear signal for BSA, as measured by UV after centrifugation, suggesting that the BSA was entirely immobilized during the polymerization process without significant leakage. These observations further proved that applying an immobilization template via the anion exchange affinity interaction approach could effectively construct binding sites within the imprinted films.

**Figure 5:**
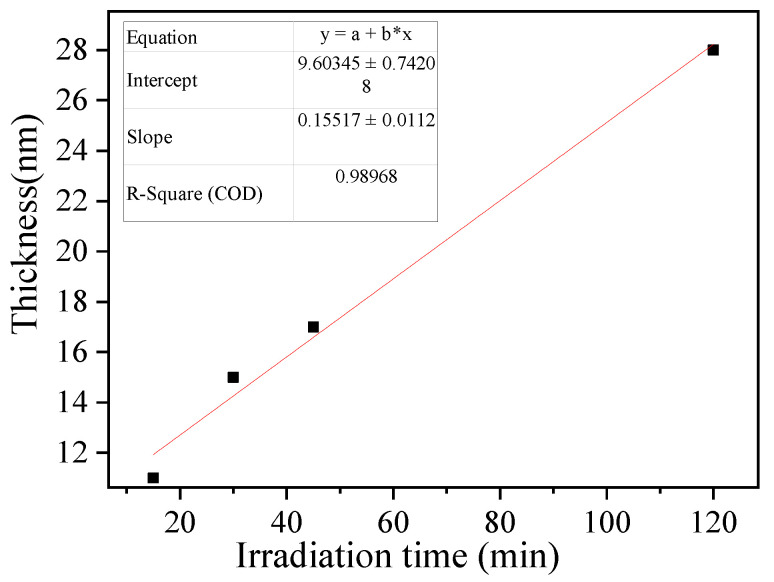
Influence of irradiation time on imprinted PVC@TAA-MIP film thickness.

Controlling the imprinted film thickness is critical for forming properly imprinted cavities with high specific recognition ability. A prolonged irradiation time results in a thick imprinted film, which in part prevents the washing of the template protein from the imprinted film and inhibits the formation of binding cavities after polymerization [44]. The thickness of the imprinted film estimated by TEM increased linearly with the irradiation time (R^2^ = 0.9897, slope = 0.1552 ± 0.0112 nm min^−1^, Figure 5). The results indicated that SI-PIMP could proceed from the dithiocarbamte groups on the substrate surface. Further, the imprinted polymer film thickness could be controlled by changing the irradiation time. The thickness of the imprinted polymer film after 30 min of irradiation was estimated to be 15 nm, which could provide the required binding cavities at the substrate surface because the theoretical diameter of the template proteins was approximately 14 nm (maximum grain size). Prolonging the SI-PIMP to 120 min significantly increased the thickness of the imprinted polymer and could potentially conceal template proteins inside the polymer. Thus, an irradiation time of 30 min was employed for the subsequent tests.

### 3.4. Elution of Template Proteins

Adding NaCl to the elution solution could break the electrostatic interaction between the BSA and imprinted polymer, whereas the addition of HAc to the elution solution could facilitate protein elution by disrupting the hydrogen bonds between the protein and polymer. Table S3 (ESI †) summarizes the effects of different NaCl–HAc ratios on the elution efficiency. Evidently, washing with high NaCl concentrations (e.g., 1 M NaCl) could enhance the elution efficiency because the anion-exchange-based affinity interaction between binding sites and BSA was affected by the anionic nature of NaCl. Moreover, it was also observed that the elution efficiency could be improved by applying an optimized HAc concentration (0.2 M HAc). This phenomenon was closely related not only to the anion-exchange-based affinity interaction of HAc but also to its ability to form hydrogen bonds. Compared to the harsh template elution conditions used in the covalent immobilization approach [21,22], the anion exchange reversible reaction enabled mild elution conditions using a NaCl–HAc solution, which was sufficient to remove over 80% of the template molecules from the imprinted films. Finally, considering the elution efficiency, washing with a solution consisting of 1 M NaCl + 0.2 M HAc was regarded as the standard elution method. 

### 3.5. Influence of NaCl Concentration on Binding Properties

**Figure 6:**
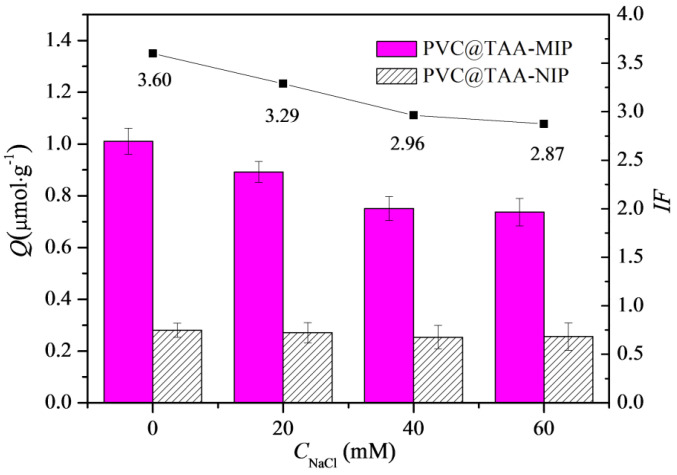
Influences of NaCl concentration on binding capacity and imprinting factor of PVC@TAA−MIP and PVC@TAA−NIP toward BSA.

The presence of NaCl in the adsorption solution could affect the adsorption ability of imprinted materials, mainly when the anion exchange mechanism is involved [24]. As shown in Figure 6, the binding capacity and imprinting factor of the PVC@TAA-MIP toward BSA gradually decreased with the increasing NaCl concentration. This observation was mainly attributed to the competition of chloride ions with the template through the anion exchange reversible reaction [45]. However, the binding capacity of PVC@TAA-NIP toward BSA showed no significant change as a function of the NaCl concentration. This revealed that the surface of the non-imprinted material lacked electrostatic interaction from residual charged moieties, because they were entirely covered after SI-PIMP. These results suggested that the non-specific binding of the template decreased significantly when using the combination of the immobilization template and surface-initiated polymerization technique. Compared to previous studies that utilized randomly oriented charged monomers to synthesize imprinted materials based on the anion exchange mechanism, the polymers reported in this study demonstrated a superior imprinting factor.

### 3.6. Binding Isotherms

**Figure 7:**
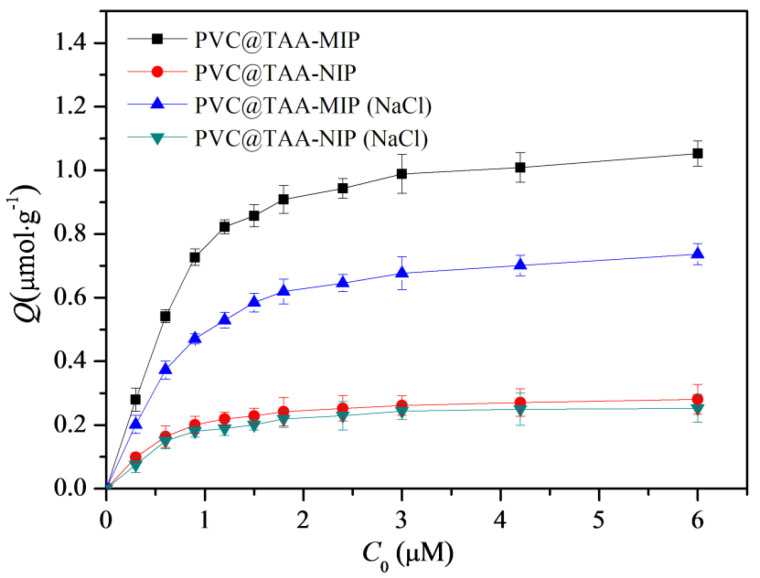
Binding isotherms of PVC@TAA−MIP and PVC@TAA−NIP toward BSA in absence or presence of NaCl.

Binding tests were implemented using PBS (pH 7.0, 10 mM) with or without 40 mM NaCl for comparison. Batch equilibrium binding tests were performed at various initial concentrations of BSA ranging from 0.3 to 6.0 µM. As shown in Figure 7, within this concentration range, the PVC@TAA-MIP exhibited much higher binding capacities than the PVC@TAA-NIP control. This suggested that molecular binding sites were produced inside the imprinted film by the involvement of the template in the polymerization process. When PBS (pH 7.0, 10 mM) was employed, the saturation binding capacities of PVC@TAA-MIP and PVC@TAA-NIP were measured to be 1.054 and 0.2806 µmol·g^−1^, respectively, achieving an imprinting factor of 3.76, which was higher than the previously reported value (2.66) using randomly oriented charged functional monomers [29]. These results confirmed the remarkable enhancement of the imprinting efficiency when using the anion exchange affinity-based immobilization imprinting strategy. When 10 mM PBS (pH 7.0) containing 40 mM NaCl was used, the saturation binding capacities of PVC@TAA-MIP and PVC@TAA-NIP were 0.7368 and 0.2518 µmol·g^−1^, respectively, achieving an imprinting factor of 2.93. This result revealed that, even with a NaCl solution with a relatively high concentration, at which anion exchange affinity-based interactions were inactivated, the imprinted materials still exhibited a high imprinting factor. This outcome confirmed the presence of a low-affinity binding mode, mainly attributed to the hydrogen bonding interaction between the amide groups of the binding sites and the amino groups of BSA in PBS (pH 7.0, 10 mM).

The Scatchard model was employed to study the binding properties of PVC@TAA-MIP [45]:

$$\frac{Q}{C}=\frac{Q_{\max}}{K_{d}}-\frac{Q}{K_{d}},$$

where *Q* and *Q*_max_ (µmol·g^−1^) are the experimental binding capacity at the equilibrium concentration and the theoretical maximum adsorption, respectively; *C* (µmol·L^−1^) is the equilibrium concentration of BSA in the bulk solution; and *K_d_* (µmol·L^−1^) is the dissociation constant. When PBS (pH 7.0, 10 mM) was employed, the Scatchard plots of the PVC@TAA-MIP deviated from a single straight line. In contrast, they consisted of two individual straight lines with different slopes. This suggests that their affinities could be approximated by two binding association constants (*K*_a_) corresponding to the high- and low-affinity sites [46]. The values of *K_d_* and *Q*_max_ were calculated to be 0.04590 µmol·L^−1^ and 0.9265 µmol·g^−1^ for high-affinity, and 0.1839 µmol·L^−1^ and 1.079 µmol·g^−1^ for low-affinity binding sites, respectively (Figure S3, ESI †). When PBS (pH 7.0, 10 mM) containing 40 mM NaCl was utilized, the Scatchard plot of the PVC@TAA-MIP was linear. Fitting the binding data using a 1:1 Langmuir model yielded an acceptable correlation coefficient (R^2^ = 0.98, Figure S3, ESI †). The value of *K_d_* was calculated to be 0.2607 µmol·L^−1^, which was very close to the binding dissociation constant of the low-affinity binding sites of PVC@TAA-MIP in the absence of NaCl. This provided further support to the hypothesis by indicating that the two affinity binding models could originate from similar interaction patterns. Thus, high-affinity binding sites might be derived from anion exchange-based affinity interactions in the absence of NaCl. Furthermore, the binding strength could be tuned to a certain extent by changing the NaCl concentration. Therefore, binding strength and efficiency adjustments can be envisioned, allowing broad applications of these polymers.

### 3.7. Binding Kinetics

**Figure 8:**
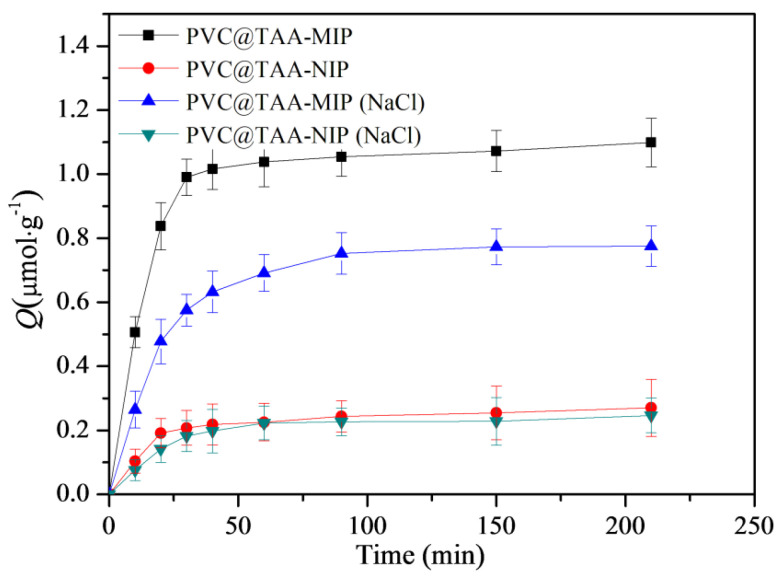
Binding kinetics of PVC@TAA−MIP and PVC@TAA−NIP toward BSA in the absence or presence of NaCl.

The binding kinetics were further studied using an initial BSA concentration of 3 µmol·L^−1^. As shown in Figure 8, when PBS (pH 7.0, 10 mM) was employed, the PVC@TAA-MIP exhibited a much stronger binding strength and a binding rate that was more than three times faster than when PBS containing NaCl was used. These results confirmed that the immobilization strategy based on anion-exchange-based affinity interaction could remarkably increase the binding rate, as previously reported by Fu et al. [24]. In addition, as previously suggested by Chen et al. [47], the ultrathin imprinted films prepared using SI-PIMP allowed the formation of homogeneous binding cavities and construction of effective binding sites situated at or close to the layer surface. This resulted in a high imprinting efficiency and fast-mass transfer rate toward the template. The binding kinetic results suggested the potential application of PVC@TAA-MIP for the removal of BSA from complex samples.

### 3.8. Binding Specificity

Selectivity tests were carried out using structurally analog proteins such as OVA, Hb, and Lys as control compounds. OVA and Hb were selected because they have isoelectric points (pIs) and molecular weight similar to those of BSA. Compared with BSA, Lys has a lower molecular weight; however, it was selected because it has the opposite charge at pH 7.0. Table 1 lists the binding capacities of PVC@TAA-MIP and PVC@TAA-NIP for these proteins at a feed concentration of 3 µM, in the absence of NaCl. As shown in Table S3, PVC@TAA-MIP exhibited significant binding selectivity toward BSA, with an imprinting factor of 3.77 and specific binding of 1.068 µmol·g^−1^, which were much higher than those toward the control proteins, indicating the specific recognition of BSA.

When PBS (pH 7.0, 10 mM) containing NaCl was employed, the PVC@TAA-MIP exhibited completely different binding capacities and imprinting factors (Table S4, ESI †), indicating that the presence of NaCl had a significant effect on the binding mechanism of PVC@TAA-MIP toward all the analytes. PVC@TAA-MIP exhibited a lower binding capacity toward OVA than that in the absence of NaCl, mainly because of the suppression of anion exchange-based high-affinity interactions in the presence of NaCl. The binding capacity of Hb, which was almost neutral at pH 7.0, showed relatively small variation despite its similar molecular weight to BSA, which could have been caused by the weak anion exchange-based affinity interaction between the Hb and imprinted polymer. Compared to OVA and Hb, Lys had a higher binding capacity, mainly because of the weak electrostatic repulsion between the positive charges of Lys and the imprinted materials in the presence of NaCl. These results indicated that PVC@TAA-MIP exhibited excellent specificity even when the anion exchange-based high-affinity interactions were suppressed, thereby expanding the application scope of this imprinted material.

**Figure 9:**
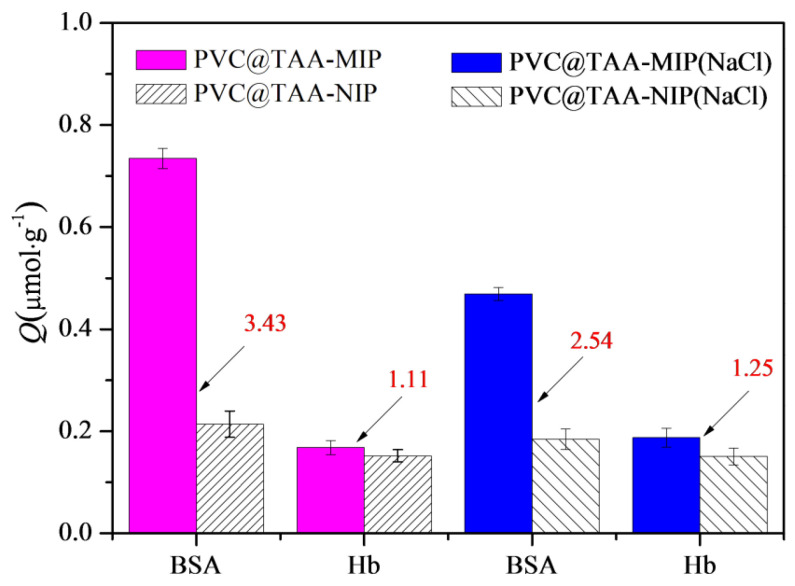
Competitive binding results for BSA and Hb to PVC@TAA−MIP and PVC@TAA−NIP in absence or presence of NaCl.

Competitive binding tests were performed using a binary protein mixture of BSA and Hb. As shown in Figure 9, PVC@TAA-MIP exhibited acceptable specific selectivity toward the template, despite the fact that a small portion of the template was not adsorbed and a portion of Hb was captured by the sorbent material. In addition, the competition parameter, which was defined as the *β*_tem_/*β*_ana_ ratio, was calculated as an index of the specific selectivity. In the absence of NaCl, the competition parameter was calculated to be 3.09, which was significantly higher than that in the presence of NaCl (2.03). Therefore, the high competition parameter resulted from the combination of anion exchange affinity-based interactions and amino group affinity-based hydrogen bondings. Furthermore, PVC@TAA-MIP provided an outstanding ability to resist interference, which is considered beneficial for BSA separation and enrichment in multi-component complicated systems.

## 4. Conclusions

In summary, this study provided a novel and versatile approach for fabricating ultrathin imprinted films using anion exchange affinity-based controllable surface imprinting. The immobilization template method overcame template protein leakage and enhanced the template immobilization efficiency. The subsequent use of SI-PIMP enabled the formation of homogeneous binding cavities and construction of effective binding sites with low non-specific binding capacity and high imprinting efficiency. By benefiting from the anion exchange mechanism, the resultant imprinted films exhibited a higher imprinting factor and faster binding rate than the reference materials. Binding tests revealed that the binding strength and selective recognition properties could be tuned to a certain extent by adjusting the NaCl concentration. Furthermore, compared to the method based on covalent immobilization, the anion exchange affinity-based interaction approach could facilitate template removal. Therefore, PVC@TAA-MIP nanoparticles are expected to be promising affinity material candidates for BSA analyses.

## Figures and Tables

**Table 1:** Imprinting efficiencies and selectivity coefficients of PVC@TAA-MIP and PVC@TAA-NIP toward BSA in absence of NaCl.

Compound	*Q* (µmol·g^−1^)		*α*	*β*
	PVC@TAA-MIP	PVC@TAA-NIP		
BSA	1.068 ± 0.0235	0.2830 ± 0.0184	3.77	—
OVA	0.3636 ± 0.0166	0.2010 ± 0.0143	1.81	2.08
Hb	0.2039 ± 0.0189	0.2295 ± 0.0125	0.89	4.24
Lys	0.0776 ± 0.0138	0.0919 ± 0.0116	0.84	4.49

## Data Availability

The data presented in this study are available on request from the corresponding author.

## Supplementary Materials

The following supporting information can be downloaded at: https://www.mdpi.com/article/10.3390/polym14102011/s1, Figure S1: FT−IR spectra of PVC, PVC@TAA and PVC@TAA−MIP; Figure S2: XPS (a) wide scan, (b) C 1s and (c) N1s of PVC@TAA-BSA nanoparticles; XPS (d) wide scan, (e) C 1s, (f) N1s and (g) S 2p of PVC@TAA−MIP nanoparticles; Figure S3: The Scatchard plots of PVC@TAA−MIP in the absence (or presence) of NaCl; Table S1: The pore structure and surface area of PVC, PVC@TAA, PVC@TAA−BSA and PVC@TAA−MIP were studied by nitrogen sorption analysis; Table S2: Influence of BSA concentration on the zeta-potentials and immobilization capacities of PVC@TAA−BSA; Table S3: Influence of washing solution on the elution efficiency of imprinted materials; Table S4: Imprinting efficiency and selectivity coefficients of PVC@TAA−MIP and PVC@TAA−NIP towards BSA in the presence of NaCl.
