# Supplementary material for: Anion Exchange Affinity-Based Controllable Surface Imprinting Synthesis of Ultrathin Imprinted Films for Protein Recognition
Source: Polymers (Basel). 2022 May 14;14(10):2011. doi: 10.3390/polym14102011 (PMC9144501; doi:10.3390/polym14102011)
Supplement: Supplementary file 1 [file polymers-14-02011-s001.zip › polymers-1699176-supplementary.pdf]

Supporting Information

Anion exchange affinity-based controllable surface imprinting synthesis of ultra-thin imprinted films for protein recognition

Renyuan Song[1]*, XiaoFeng Yu[1,2], Muxin Liu[1], Xiaoling Hu[3], Shengqing Zhu[1]

[1] School of Materials and Chemical Engineering, Bengbu University, Bengbu 233030, P. R. China; schemistry01@163.com (M.L.); yuxfchem@126.com(S.Z.)

[2] Bengbu Product Quality and Inspection Institute,Bengbu, Anhui 233030, P. R. China ; yuxfchemistry@163.com (X.Y.)

[3] School of Chemistry and Chemical Engineering, Northwestern Polytechnical University, Xi'an 710129, P. R. China; shichemistry@126.com(X.H.)

* Correspondence: feiyu@mail.ustc.edu.cn (R.S.)

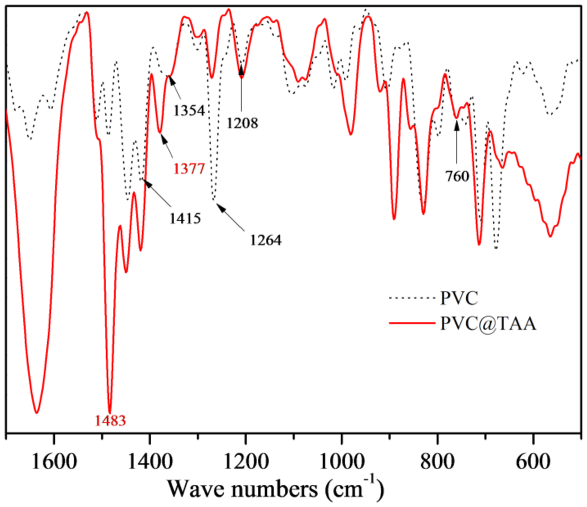

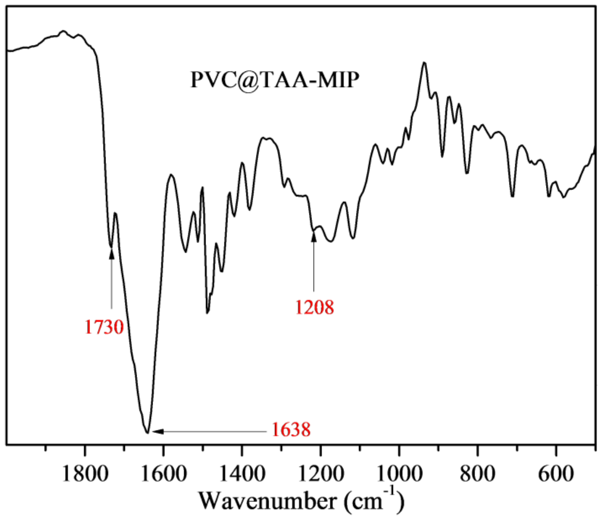

Figure S1. FT-IR spectra of PVC, PVC@TAA and PVC@TAA-MIP.

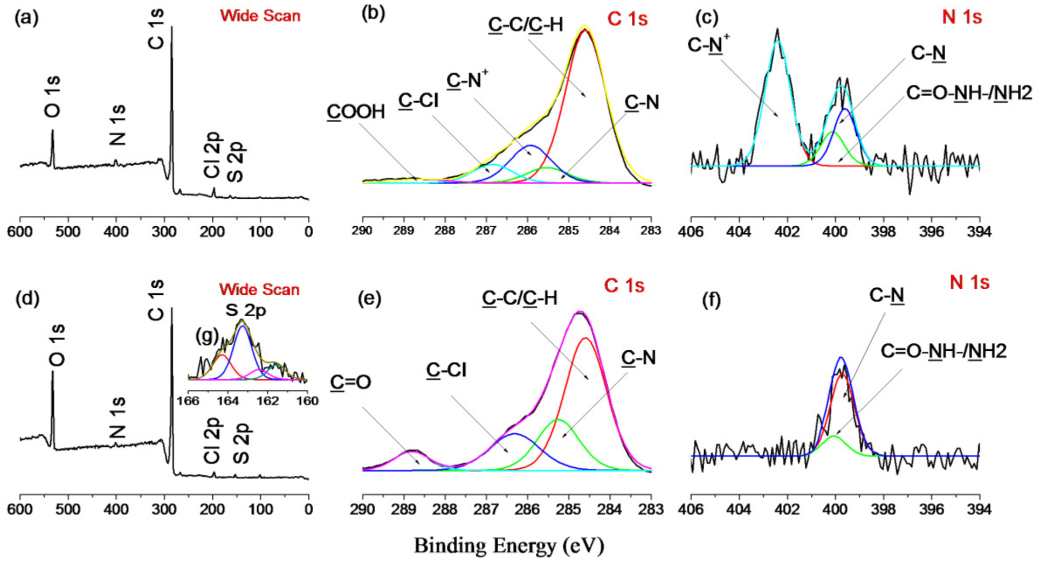

Figure S2. XPS (a) wide scan, (b) C 1s and (c) N1s of PVC@TAA-BSA nanoparticles; XPS (d) wide scan, (e) C 1s, (f) N1s and (g) S 2p of PVC@TAA-MIP nanoparticles.

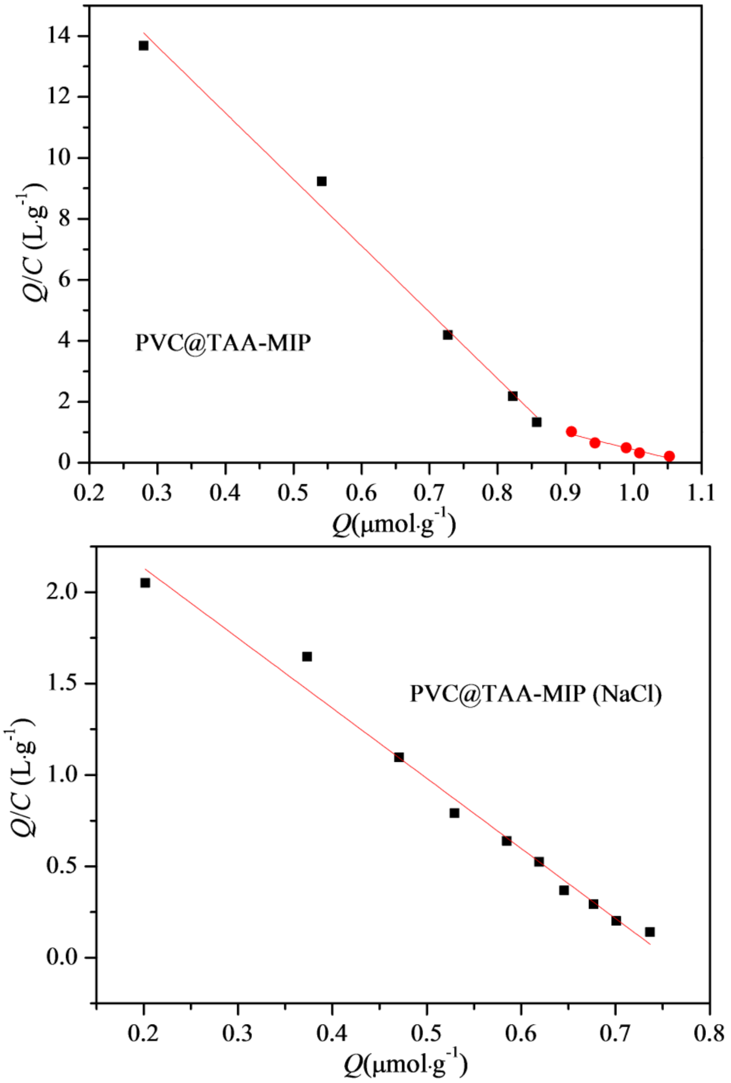

Figure S3. The Scatchard plots of PVC@TAA-MIP in the absence (or presence) of NaCl.

Table S1. The pore structure and surface area of PVC, PVC@TAA, PVC@TAA-BSA and PVC@TAA-MIP were studied by nitrogen sorption analysis.

Sorbents	S ($m^2 \cdot g^{-1}$)	V_p ($cm^3 \cdot g^{-1}$)	d_p (nm)
PVC	34.87	–	–
PVC@TAA	119.63	1.121	23.43
PVC@TAA-BSA	83.41	1.435	14.63
PVC@TAA-MIP	104.59	1.638	9.57

Table S2. Influence of BSA concentration on the zeta-potentials and immobilization capacities of PVC@TAA-BSA.

Samples concentration (μM)	Zeta-potential (mV)	Immobilization capacity ($\mu mol \cdot g^{-1}$)
1.2	21.19 ± 0.40	0.234 ± 0.0089
2.4	13.67 ± 0.32	0.564 ± 0.0171
3.6	6.28 ± 0.21	0.768 ± 0.0234
4.8	-11.29 ± 0.55	0.992 ± 0.0363
6.0	-30.29 ± 0.74	1.136 ± 0.0527

Table S3. Influence of washing solution on the elution efficiency of imprinted materials.

Washing solution	Elution efficiency
0.5 M NaCl	57.6 %
1 M NaCl	71.4 %
1 M NaCl + 0.1 M HAc	79.1 %
1 M NaCl + 0.2 M HAc	82.2 %
1 M NaCl + 0.4 M HAc	83.6 %

Table S4. Imprinting efficiency and selectivity coefficients of PVC@TAA-MIP and PVC@TAA-NIP towards BSA in the presence of NaCl.

Compound	Q ($\mu mol \cdot g^{-1}$)		α	β
	PVC@TAA-MIP (NaCl)	PVC@TAA-NIP (NaCl)		
BSA	0.7662 ± 0.0201	0.2574 ± 0.0102	2.98	–
OVA	0.2284 ± 0.0162	0.1967 ± 0.0188	1.16	2.57
Hb	0.1752 ± 0.0230	0.1693 ± 0.0156	1.03	2.89
Lys	0.1190 ± 0.0241	0.1064 ± 0.0079	1.12	2.66
